# Induction of Chaperone Synthesis in Human Neuronal Cells Blocks Oxidative Stress-Induced Aging

**DOI:** 10.32607/actanaturae.27531

**Published:** 2025

**Authors:** E. A. Dutysheva, L. S. Kuznetcova, I. A. Utepova, B. A. Margulis, I. V. Guzhova, V. F. Lazarev

**Affiliations:** Institute of Cytology RAS, St. Petersburg, 194064 Russian Federation; Ural Federal University named after the first President of Russia B. N. Yeltsin, Yekaterinburg, 620002 Russian Federation; I. Ya. Postovsky Institute of Organic Synthesis, Ural Branch of the Russian Academy of Sciences, Yekaterinburg, 620108 Russian Federation

**Keywords:** oxidative stress, senescence, chaperones, pyrrolylazines, apoptosis, neuroprotection

## Abstract

Oxidative stress accompanies many pathologies that are characterized by neuronal degradation leading to a deterioration of the disease. The main causes are the disruption of protein homeostasis and activation of irreversible processes of cell cycle disruption and deterioration of cellular physiology, leading to senescence. In this paper, we propose a new approach to combating senescence caused by oxidative stress. This approach is based on the use of a low-molecular inducer of chaperone synthesis, one of the cell protective systems regulating proteostasis and apoptosis. We present data demonstrating the ability of the pyrrolylazine derivative PQ-29 to induce chaperone accumulation in human neuronal cells and prevent oxidative stress-induced aging.

## INTRODUCTION

Oxidative stress accompanies the majority of disorders characterized by neuronal degradation, including neurodegenerative diseases, traumatic brain injury, stroke, etc. In these disorders, the production of reactive oxygen species (ROS) in neuronal cells causes damage to proteins, lipids, and DNA and thus provokes cell senescence, which increases the risk of concomitant diseases.

Mitochondria are the main ROS source and target in the cell. ROS can cause the collapse of the mitochondrial membrane potential, the disruption of mitochondrial ultrastructure, and ATP depletion [1]. Damage to mitochondria can lead to necrosis and apoptosis. In addition, oxidative stress and mitochondrial malfunction can activate the p53/p21 and Rb/p16 pathways [2]. Both pathways increase the expression and activity of senescence-associated β-galactosidase. Therefore, combating the effects of cell-damaging oxidative stress is an important part of treating the majority of neurodegenerative diseases.

Heat shock proteins (HSP, chaperones) are important neuroprotective factors. These proteins play a significant role in preventing many types of cell death by targeting and destroying damaged proteins in the cell. For example, chaperone Hsp70 can prevent apoptosome formation, interact with the apoptosis-inducing factor (AIF) and pro-apoptotic protein Bim, and deactivate caspases 3 and 7 [3, 4, 5].

Another chaperone, Hsp90, also suppresses the activation of cell death signaling pathways. Hsp90 was shown to prevent apoptosome formation by binding to Apaf-1 and further inhibiting the oligomerization of the latter and its recruitment of caspase-9 [6]. It is important to note that both Hsp70 and Hsp90 bind denatured, misfolded proteins – including those misfolded due to excessive oxidation – and prevent their assembly into oligomers and aggregates [7].

Other important proteins required for the proper functioning of the chaperone machinery are co-chaperones. They are polypeptides containing the J domain, such as the Hsp40 protein. Co-chaperones regulate the formation of complexes between Hsp70 and client proteins, thus recognizing and degrading denatured and oxidized proteins [8].

In this context, it becomes interesting to study the potential chemical compounds might possess to stimulate the production of heat shock proteins for the protection of the nervous system. Compounds that are capable of inducing chaperone accumulation in cells have demonstrated their effectiveness in such disease models as Parkinson’s [9], Alzheimer’s [10], secondary damage after traumatic brain injury [11], and many others [12]. We have previously established that some pyrrolylazine derivatives can activate chaperone synthesis and accumulation, exerting a therapeutic effect in an *in vitro *model of Alzheimer’s disease [13]. The PQ-29 derivative (3-(5-phenyl-1H-pyrrol-2-yl) quinoxalin-2(1H)-one) proved the most effective in this regard. In this work, we studied the ability of this compound to stave off oxidative stress-induced senescence in human neuronal cells.

## EXPERIMENTAL

**Neuronal cells**

To confirm the chaperone-inducing and neuroprotective effects of pyrrolylazines, we used human dental pulp-derived mesenchymal stem cells (MSC-DP) as previously described [14]. MSC-DP cells were obtained from the “Vertebrate Cell Culture Collection” supported by the Ministry of Education and Science of the Russian Federation (Agreement No. 075-15- 2021-683). The cells were cultured in a DMEM/F12 medium (Gibco, USA) supplemented with 10% fetal bovine serum (FBS; Gibco), 100 U/ml penicillin, and 0.1 mg/ml streptomycin (BioloT Ltd., Russia) at 37°C and 5% CO_2_.

The cells were reprogrammed into neuronal-phenotype cells (MSC-Neu) by incubation in a Neurobasal medium (BioinnLabs, Russia) supplemented with Neuromax (PanEco, Russia), 3% FBS, 100 U/ml penicillin, and 0.1 mg/ml streptomycin (PanEco) for 5 days. The neuronal phenotype was verified by analyzing the expression of a panel of mature neuron markers [15, 16], including β3-tubulin, NeuN, MAP2, synaptophysin (SYP), PSD95, and NeuroD1 by real-time RT-PCR.

**RNA isolation and real-time PCR**

**Table 1:** The primers used in the study

Gene	Primer, nucleotide sequence
Actin	F – 5’-TCAATGTCCCAGCCATGTATGT-3’
	R – 5’-GTGACACCATCTCCAGAGTCC-3’
NeuN	F – 5’-CAAGGACGGTCCAGAAGGAG-3’
	R – 5’-GGTAGTGGGAGGTGAGGTCT-3’
MAP2	F – 5’-GGAGGGCGCTAAGTCCG-3’
	R – 5’-AAAATCTGGGCGCAGAAACTG-3’
NeuroD1	F – 5’-TCTTCCACGTTAAGCCTCCG-3’
	R – 5’- CCATCAAAGGAAGGGCTGGT-3’
β3-tubulin	F – 5’-CCATGAAGGAGGTGGACGAG-3’
	R – 5’-ACGTTGTTGGGGATCCACTC-3’
Syp	F – 5’-CTTCGCCATCTTOGCCTTTG-3’
	R – 5’-TCACTCTCGGTCTTGTTGGC-3’
PSD95	F – 5’-GGATATGTGAACGGGACCGA-3’
	R – 5’-AAGCCCAGACCTGAGTTACC-3’
p16	F – 5’-ATAGTTACGGTCGGAGGCCG-3’
	R – 5’-CACGGGTCGGGTGAGAGTG-3’
p21	F – 5’-CTCAGAGGAGGCGCCATGT-3’
	R – 5’-CGCCATTAGCGCATCACAG-3’

RNA was isolated using an ExtractRNA kit (JSC Evrogen, Russia). Reverse transcription was conducted using a MMLV RT kit (JSC Evrogen) according to the manufacturer’s instructions. RT-PCR was performed using the CFX96 real-time PCR detection system (BioRad, USA) and qPCRmix-HS SYBR kit (JSC Evrogen) according to the manufacturer’s protocol. PCR amplicon specificity was confirmed by melting curve analysis. Primer sequences are presented in *Table 1*. All primers were synthesized by JSC Evrogen. PCR parameters were as follows: 5 min pre-denaturation at 95°C followed by 40 cycles of 30 s at 95°C, 30 s at 65°C, and 30 s at 70°C. The fold change was analyzed using the BioRadCFX software.

**Aging analysis**

The activity of β-galactosidase in MSC-DP and MSC-Neu cells was assessed using a Beta-Glo assay system (Promega, UK) according to the manufacturer’s instructions. Luminescence was measured on a Varioskan LUX microplate reader (Thermo Fisher Scientific, USA).

**Electrophoresis and western blot analysis**

MSC-Neu cells were treated with 100 μM hydrogen peroxide for 2 h and incubated with PQ-29 at concentrations of 0.5, 2, 8, and 300 μM for 1 and 2 h. The cells were lysed; lysates were used for electrophoresis and blotting analysis according to the previously described protocol [17]. Antibodies against Hsp40 (clone J32), Hsp70 (clone 3C5) [18], and Hsp90 (Thermo Fisher Scientific) were used for the analysis. Anti-tubulin antibodies (Thermo Fisher Scientific) were used as a loading control. Horseradish peroxidase (HRP)-conjugated goat anti-mouse and anti-rab- bit antibodies (Repertoire, Russia) were used as secondary antibodies. Band intensities were calculated in arbitrary units (A.U.) using the TotalLab Quant 1.0 software (TotalLab, Gosforth, UK). The data were normalized to the mean intensity of tubulin staining.

**Cytotoxicity analysis**

The cytotoxic effects of PQ-29 were evaluated using the Mosmann dehydrogenase activity MTT assay [19]. LC50 was determined for PQ-29 in MSC-Neu cells. The cells were incubated with PQ-29 at a concentration range of 0.05 to 1 000 μM. The MTT test was conducted 48 h after incubation. Each experiment was performed in quadruplicate.

To confirm necrosis and apoptosis, the cells were placed in a 96-well plate and treated with 5 mg/ml ethidium bromide and 5 mg/ml acridine orange in phosphate-buffered saline (PBS). The stained cells were then examined on a Zeiss Axioscope (Carl Zeiss, Germany).

**Statistical analysis**

The mean ± standard deviation was calculated. Data were processed using the non-parametric Mann– Whitney test and the GraphPad Prism 8 software. Each experiment was conducted in at least a triplicate. Differences were considered statistically significant at *p* < 0.05.

## RESULTS

**Fig. 1:**
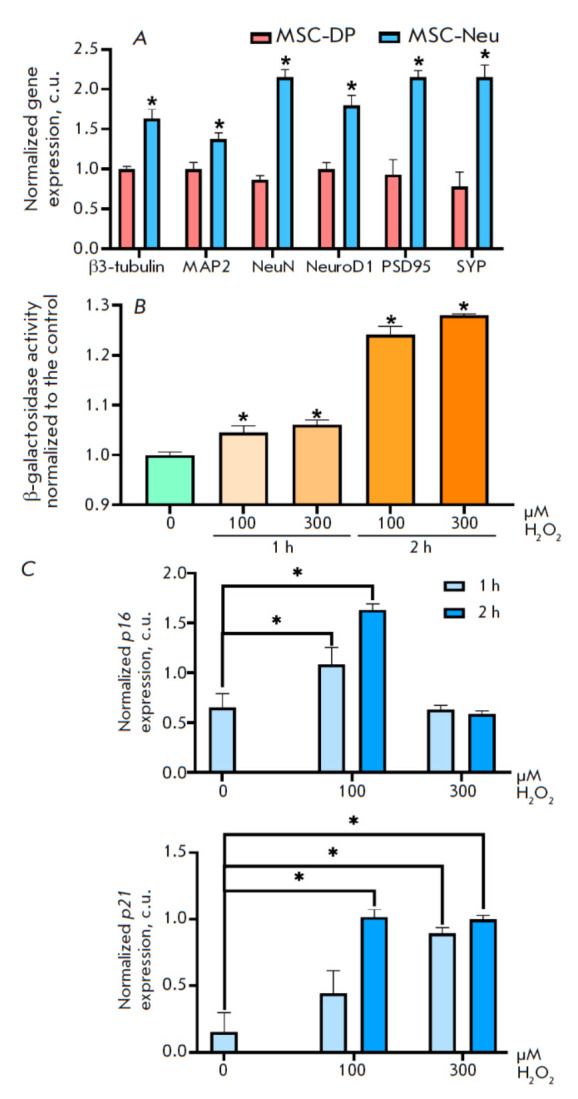
Hydrogen peroxide induces senescence in reprogrammed human MSC-Neu neurons. (A) Expression of neuronal markers in MSC-DP (before differentiation) and MSC-Neu (after differentiation) cells. Actin mRNA was used as a control. (B) Incubation of MSC-Neu cells with 100 and 300 μM hydrogen peroxide for 1 and 2 h increases β-galactosidase activity. (C) Incubation of MSC-Neu cells in the presence of 100 and 300 μM hydrogen peroxide for 1 and 2 h increases the p16 and p21 mRNA levels. Data represent the mean ± standard deviation of three separate experiments; differences are significant at *p < 0.05 (determined using the Mann–Whitney test)

At the first stage of the study, we tested the human model of oxidative stress-induced neuronal aging. For this, we used dental pulp-derived mesenchymal stem cells (MSC-DP) reprogrammed into the neuronal phenotype (MSC-Neu). To confirm the MSC-Neu neuronal phenotype after cell differentiation, we analyzed the following neuronal markers by RT-PCR: β3-tubulin, MAP2, SYP, NeuroD1, PSD95, and NeuN. The RT-PCR showed a significant increase in the mRNA levels of the studied genes after differentiation. The expression of mature neuron markers (namely, SYP, NeuroD1, PSD95, and NeuN) increased approximately threefold (*Fig. 1A*). The expression of early neuronal markers (β3-tubulin and MAP2) also increased, although insignificantly compared to that of mature neuron markers: approximately 1.4- to 1.5-fold.

We analyzed the ability of hydrogen peroxide to induce senescence in human neuronal cells. For this, MSC-Neu cells were cultured in either 100 or 300 μM hydrogen peroxide for 1 and 2 h. Next, the activity of β-galactosidase, a common senescence marker, was determined using a Beta-Glo assay system (*Fig. 1B*). Incubation of MSC-Neu cells with 100 and 300 μM hydrogen peroxide for 1 h resulted in an increase in β-galactosidase activity by 4.4 and 6.2%, respectively. Incubation of MSC-Neu cells in the presence of 100 and 300 μM hydrogen peroxide for 2 h led to an increase in β-galactosidase activity by 24.2 and 28.1%, respectively.

To confirm that the change in β-galactosidase activity is relevant, p16 and p21 expressions were analyzed. These proteins play an important role in two key senescence-initiating pathways. A RT-PCR analysis demonstrated that cell incubation with 100 μM hydrogen peroxide for 1 h resulted in a 1.68- and 2.93-fold increase in the p16 and p21 mRNA levels, respectively. Cell incubation under the same conditions for 2 h resulted in a 2.55- and 6.78-fold increase in the p16 and p21 mRNA levels, respectively (*Fig. 1C*). The use of higher hydrogen peroxide concentrations did not enhance p16 and p21 mRNA expression, which is, apparently, due to high toxicity. In further experiments on modeling oxidative stress-induced senescence, we incubated MSC-Neu cells in the presence of 100 μM hydrogen peroxide for 2 h.

**Fig. 2:**
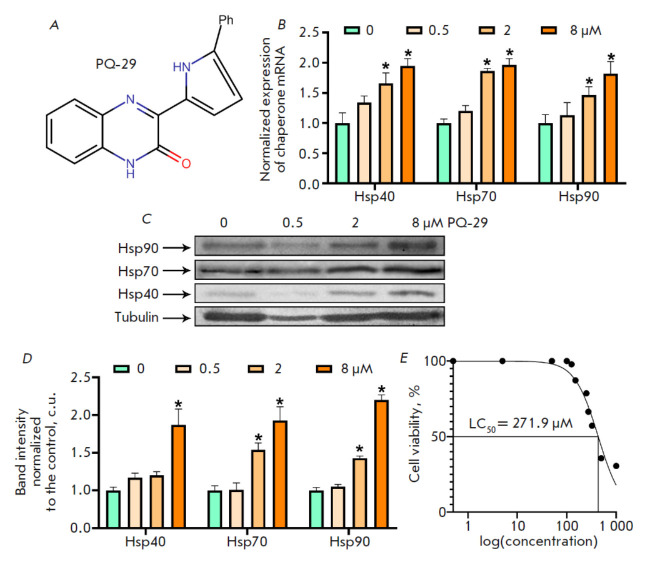
PQ-29 at non-toxic doses increases chaperone levels in MSC-Neu under oxidative stress. (A) PQ-29 structural formula. (B) Chaperone expression in MSC-Neu cells after 6-h incubation with PQ-29. (C) Western blot analysis of the Hsp90, Hsp70, and Hsp40 levels in MSC-Neu cell lysates incubated with PQ-29 at the indicated concentrations for 24 h. Tubulin was used as a loading control. Representative images are provided. (D) Band intensity ratios of Hsp90, Hsp70, Hsp40, and tubulin normalized to the control cells. (E) PQ-29 LC50 in MSC-Neu cells under oxidative stress. Data represent the mean ± standard deviation of three separate experiments; the observed differences are statistically significant at *p < 0.05 (determined using the Mann–Whitney test)

The next stage of our study was to investigate the ability of PQ-29 (the structural formula is shown in *Fig. 2A*) to activate chaperone synthesis and accumulation in neuronal cells aged under oxidative stress conditions. We had previously established the ability of PQ-29 to induce chaperone production in neuronal cells. However, it was necessary to confirm that PQ-29 can also affect aging cells. We conducted RT-PCR to evaluate the chaperone mRNA level in MSC-Neu cells aged under oxidative stress by incubation with PQ-29 for 6 h. The expression of major inducible chaperones, i.e., Hsp40, Hsp70, and Hsp90, was found to increase after PQ-29 treatment of cells aged under oxidative stress. The use of 8 μM PQ-29 resulted in 1.95-, 1.97-, and 1.82-fold increases in the Hsp40, Hsp70, and Hsp90 mRNA levels, respectively (*Fig. 2B*). Further, the chaperone level was assessed by western blot analysis in MSC-Neu cells incubated in the presence of PQ-29 for 4 h. The addition of 8 μM PQ-29 to the cells resulted in a 1.87-, 1.93-, and 2.2-fold increase in Hsp40, Hsp70, and Hsp90 mRNAs, respectively (*Fig. 2C,D*).

We had previously established that PQ-29 has low cytotoxicity [13]; however, we had to also confirm that the cytotoxicity effect would not rise in oxidative stress-aged cells. For this, we determined LC50 of PQ-29 in MSC-Neu cells aged under oxidative stress by MTT analysis (*Fig. 2E*). The LC50 was found to be 271.9 μM. Thus, the PQ-29 concentrations used did not have a significant cytotoxic effect on the aged cells.

**Fig. 3:**
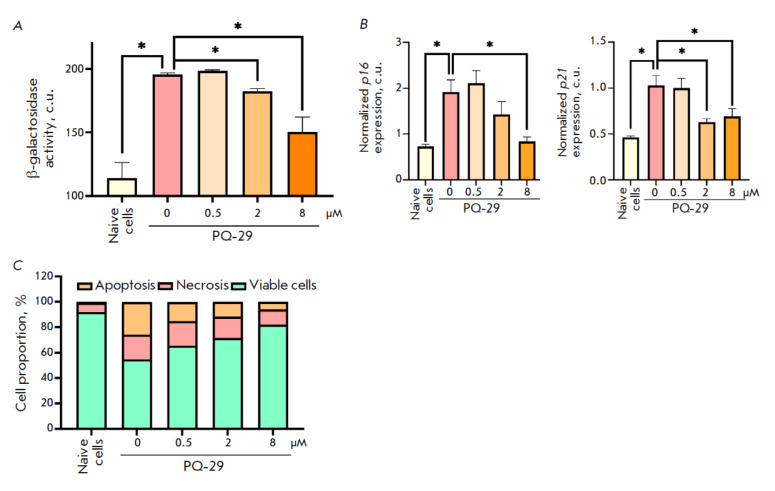
PQ29 prevents oxidative stress-induced senescence in MSC-Neu cells. (A) β-galactosidase activity in MSC-Neu cells after 24-h incubation in the presence of PQ-29 and senescence induction by hydrogen peroxide. A mammalian Beta-Glo assay system was used. (B) Expression of p16 (left panel) and p21 (right panel) in MSC-Neu cells after 24-h incubation with PQ-29 and senescence induction by hydrogen peroxide. (C) Acridine orange staining. The proportion of viable, apoptotic, and necrotic MSC-Neu cells after 24-h incubation in the presence of PQ-29 and senescence induction by hydrogen peroxide is presented. Data represent the mean ± standard deviation of three separate experiments; the observed differences are statistically significant at *p < 0.05 (determined using the Mann–Whitney test)

At the final stage of the study, we investigated the ability of PQ-29 to prevent oxidative stress-induced aging and the degradation of neurons. MSC-Neu cells subjected to oxidative stress-induced aging were cultured at different PQ-29 concentrations for further evaluation of β-galactosidase activity (using a Beta-Glo assay system) and cell viability (by staining with acridine orange). PQ-29 at concentrations of 2 and 8 μM reduced the increase in the β-galactosidase activity due to oxidative stress by 9.4 and 24.3%, respectively (*Fig. 3A*). We next analyzed the gene expression of the common senescence markers p16 and p21 in aging MSC-Neu cells in the presence of PQ-29. PQ-29 at a concentration of 8 μM was found to reduce the increase in p16 expression induced by oxidative stress by 78.3% (*Fig. 3B*, left panel). Both 2 and 8 μM PQ-29 supressed growth in p21 expression: by 54.7 and 47.8%, respectively (*Fig. 3B*, left panel). Finally, using acridine orange staining, we determined the proportion of cells subjected to oxidative stress-induced aging that underwent either apoptosis or necrosis, and we confirmed the ability of PQ-29 to prevent cell death. PQ-29 at concentrations of 2 and 8 μM prevented the development of both necrosis and apoptosis in neuronal cells. The proportion of necrotic cells decreased from 19.6 to 17.1 and 12.2% (in the presence of 2 and 8 μM PQ-29, respectively), while the proportion of apoptotic cells decreased from 25.6 to 11.4 and 5.8% (when using 2 and 8 μM PQ-29, respectively). Thus, the use of 2 and 8 μM PQ-29 resulted in an increase in the proportion of naive cells from 54.7 to 71.5 and 82%, respectively (*Fig. 3C*). These data indicate that PQ-29 can prevent oxidative stress-induced cell aging.

## DISCUSSION

**Fig. 4:**
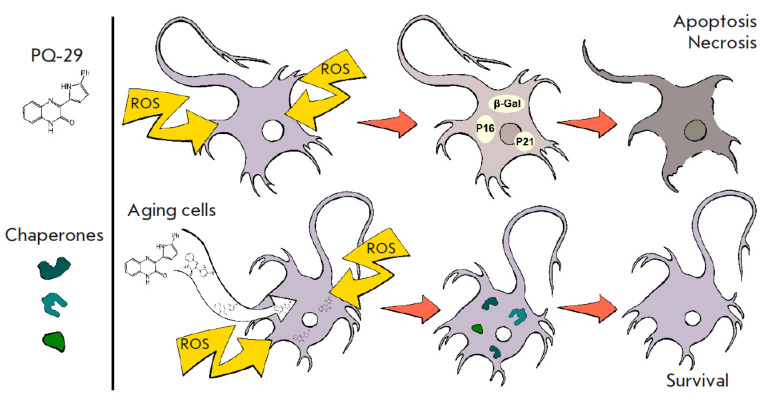
Illustration of the principle of action of low-molecular-weight chaperone inducers to protect neuronal cells from oxidative stress

The lack of an effective response from the antioxidant cell system to oxidative stress is known to result in various pathologies. This is partially due to the inability of protein homeostasis systems to cope with the increasing number of damaged and mutated proteins [20]. Another negative effect of oxidative stress is the triggering of irreversible processes that disrupt the cell cycle and affect cellular physiology, leading to senescence. One of the mechanisms that protect cells, including neurons subjected to oxidative stress, involves, apart from antioxidants, chaperone synthesis inducers, which can enhance neuronal resistance to oxidative stress (*Fig. 4*). Such studies have already been conducted. For instance, the chaperone synthesis inducer U133 was shown to increase the resistance of C6 rat glioblastoma cells to ROS [17]. In addition, activation of chaperone synthesis reduces the proteotoxic load on cells associated with oxidative stress [21]. At the same time, delayed negative processes, including activation of senescence mechanisms, represents another important risk affecting neuronal function, in addition to a decrease in acute toxicity due to oxidative stress; namely, the oxidation of proteins and lipids and activation of apoptosis. Chaperones are known to prevent senescence activation through the p53/p21 and Rb/p16 signaling pathways [2]. However, the studies that have reported this regulation were conducted in cancer cells and cannot be considered relevant to neurodegenerative processes. Furthermore, chaperone expression in neuronal cells usually decreases with pathology progression; in particular, this phenomenon is found in traumatic brain injury, stroke, and Alzheimer’s disease.

Chaperone synthesis inducers have been studied as promising neuroprotective drugs for a long time; the chaperone inducer arimoclomol is currently undergoing clinical trials [22]. However, there are no data on the effect of inducers on senescence. In this work, we propose PQ-29 as an agent capable of activating the production of the key chaperones Hsp70 and Hsp90 and co-chaperone Hsp40. The use of PQ-29 made it possible to not only inhibit the delayed cytotoxic effect of oxidative stress, but also to prevent neuronal cell senescence initiation in the presence of ROS. We would also like to note that LC50 of PQ-29 in old neurons was lower than that in neurons that had not undergone oxidative stress: 271 and 494 μM, respectively [10]. This indicates that the resistance of cells subjected to oxidative stress-induced aging decreases due to the effect of chemical agents.

We previously synthesized some pyrrolylazine compounds (including PQ-29) and established their ability to induce heat shock protein synthesis and exert a neuroprotective effect in Alzheimer’s disease and traumatic brain injury [11, 13, 23]. In addition, the ability of pyrrolylazine derivatives to induce Hsp70 production was confirmed in both young and old reprogrammed human MSCWJ-Neu neurons. In the present study, we have established the ability of the pyrrolylazine derivative PQ-29 to prevent oxidative stress-induced aging (*Fig. 4*). Taken together, these data allow us to conclude that these compounds possess a pronounced neuroprotective activity.

## Abbreviations

ROS: reactive oxygen species
HSP: heat shock protein
ATP: adenosine triphosphate
AIF: apoptosis inducing factor
HRP: horseradish peroxidase
PBS: phosphate-buffered saline
FBS: fetal bovine serum
SYP: synaptophysin
